# Interest in Co-located Reproductive and Sexual Health Services Among Women and Men Receiving Medication for Opioid Use Disorder in an Outpatient Treatment Clinic

**DOI:** 10.3389/fpsyt.2022.910389

**Published:** 2022-07-06

**Authors:** Jonathan J. K. Stoltman, Laura R. Lander, Julie H. Patrick, Mishka Terplan, Hendrée E. Jones

**Affiliations:** ^1^Opioid Policy Institute, East Grand Rapids, MI, United States; ^2^Healthy Aging Lab, Department of Psychology, West Virginia University, Morgantown, WV, United States; ^3^Psychology Department, Grand Valley State University, Allendale, MI, United States; ^4^Department of Behavioral Medicine and Psychiatry, West Virginia University School of Medicine, Rockefeller Neuroscience Institute, Morgantown, WV, United States; ^5^Department of Neuroscience, West Virginia University School of Medicine, Morgantown, WV, United States; ^6^Friends Research Institute, Baltimore, MD, United States; ^7^UNC Horizons and Department of Obstetrics and Gynecology, University of North Carolina at Chapel Hill, Chapel Hill, NC, United States; ^8^Department of Psychiatry and Behavioral Sciences and Obstetrics and Gynecology, School of Medicine, Johns Hopkins University, Baltimore, MD, United States

**Keywords:** opioid use disorder, buprenorphine, contraceptives, STI/STD, rural health

## Abstract

**Introduction:**

Reproductive and sexual health (RSH) are core components of comprehensive care, yet often omitted in addiction treatment. We characterize knowledge of and interest in RSH services and contraceptive method awareness and use in a rural, Appalachian outpatient clinic.

**Materials and Methods:**

Between September 2016 and April 2018, a convenience sample of 225 patients receiving treatment for opioid use disorder at an outpatient buprenorphine/naloxone clinic was collected. Participants completed a cross-sectional RSH survey that included demographics, interest in RSH service integration, contraceptive use, and contraceptive knowledge.

**Results:**

A total of 212 people (126 non-pregnant women, 29 pregnant women, and 57 men) completed the survey of whom 45.8% indicated interest in adding RSH services. Services of interest include regular physical exams (44.8%), STI/STD testing (41.0%), and contraception education and administration (38.2%). There were no significant differences between interest in co-located services between women and men (*P* = 0.327). Current contraceptive use was low (17.9–30.9%) among women and men. Contraceptive method awareness was 43.3% for high efficacy methods and 50.0% for medium efficacy methods. Women and currently pregnant women knew more total, high, and medium efficacy contraceptive method than men (*P* = 0.029).

**Discussion:**

Both women and men in this sample are interested in co-located RSH services. Current contraceptive use was low among participants. Contraceptive knowledge was lower among men compared to women, and generally low. Providing co-located RSH services may facilitate RSH education, contraceptive method uptake, and promote engagement across various RSH domains.

## Introduction

Reproductive health addresses the reproductive processes, functions, and system at all stages of life (e.g., contraceptive counseling) while sexual health is a state of physical, mental, and social well-being in relation to sexuality (e.g., sexual functioning) (1). Although reproductive and sexual health (RSH) is recognized as a key component of holistic medicine, integration of RSH services is lacking in opioid use disorder (OUD) treatment, outside of the attention to the needs of pregnant women with OUD (2). A goal of the Affordable Care Act was to break down the barriers between care systems (3), however, system-level barriers continue, especially for patients receiving OUD treatment (4).

Previous research has shown some interest in women’s health services being integrated into addiction treatment facilities (5); however, the degree to which RSH services have been integrated into OUD treatment facilities is low although robust national data are lacking. A recent survey focused on the RSH needs of reproductive-age women assessed opioid treatment programs in North Carolina and found that clinic directors see a need for co-located RSH services; however, only approximately 50% provided HIV testing and contraceptives (2). Further, non-pregnant women receiving medication for opioid use disorder (MOUD) show high rates of RSH service utilization when such services are offered (6).

Relatively little data exist regarding overall RSH needs among patients with OUD, especially among men. One reason for this discrepancy may be the focus on the high unintended pregnancy rates experience by pregnant women with OUD. In a landmark treatment trial, MOUD participants had double the rate of unintended pregnancy (86%), compared to the general population (31–47%) (7). The high rates of unintended pregnancy among pregnant women with OUD may be traced back to limited contraceptive use (8) and barriers to accessing RSH services (9). For example, women with substance use disorders are 25% less likely than the general population to use contraceptives (8) and most frequently endorsed condom use (62%), while high efficacy contraceptive methods, such as intrauterine devices (8%), were less frequently endorsed. Condom use is even lower (approximately 20%) among men receiving MOUD (10, 11). This mismatch between the high unintended pregnancy rate and low use of high efficacy contraception could benefit from a broader understanding of the challenges faced by patients trying to achieve their RSH goals.

By broadening the understanding of RSH from a focus on pregnant women with OUD to one that includes women (non-pregnant and pregnant) and men with OUD we can better understand the extent RSH services are desired and what RSH services to co-locate. This study aims to: (1) describe interest in RSH services among people receiving MOUD; (2) characterize patient contraceptive method use, knowledge, acceptability, and barriers to use; and (3) determining if gender differences are present in RSH domains.

## Materials and Methods

This cross-sectional survey was conducted at a single clinic between September 2016 and April 2018. The Comprehensive Opioid Addiction Treatment (COAT) Clinic at West Virginia University serves patients with OUD receiving MOUD (at the time, exclusively buprenorphine/naloxone medication) and a mix of group and individual therapy sessions (12). All patients over the age of 18 were potential participants in this study. The West Virginia University Institution Review Board approved this study. Data were collected in accordance with the Declaration of Helsinki (2013).

### Procedure

To collect this convenience sample, potential participants were approached in the therapy group meeting space after the group therapy session was completed. After providing a brief study description, those who were interested in the study stayed in the room and completed consent. After consent, each participant was provided a confidential ID to link surveys across study sessions. The linking document that contained participant IDs was also used to verify if the participant was already enrolled in the study. It is estimated that our recruitment efforts reached approximately 75% of the patients enrolled in the clinic during the study timeframe of whom over 50% participated in the study. The recruitment methodology, however, prevented careful evaluation of how many participants were approached or refused study participation.

Surveys were individually completed in the group setting using a 7” Amazon Fire capacitive touch screen tablets and REDCAP online survey software (13). Trained research staff spent time with each participant adjusting the font size and orienting them to the touch-screen device. Participants were provided a rubber-tipped non-active stylus to interact with the tablet computer if needed (e.g., long fingernails). Participants received a $10 gift card to a national retailer following survey completion. Research staff remained in the room to answer questions and troubleshoot device issues.

### Measures

A multidisciplinary team (psychology, social work, public health, addiction medicine, obstetrics, and gynecology) developed the survey. The survey was piloted with 50 women to test the technology, determine ease of use, and assess overall survey length and acceptability. The final survey version took 15 min to complete and had a Flesch-Kincaid score equivalent to a sixth grade reading level. Colloquial terms (e.g., rubbers) and brand names (e.g., Trojan) were used, when possible, to enhance comprehension and compliment the medical terminology included in the survey. All questions were asked of both women and men with gender-specific tailoring when relevant. Any question could be skipped as deemed necessary by the participant. See supplement for the full questionnaire.

#### Reproductive Health

Survey questions related to RSH included past year sexual activity, sexual partner’s gender, frequency of emergency contraception (“Plan B”), and whether they would ever consider ending a pregnancy early. Gender was assessed *via* self-report. Pregnancy intention was assessed with the One Key Question format. The One Key Question (OKQ) “would you like to get pregnant in the next year?” was developed as a concise way to determine pregnancy interest and provide a gateway to a more comprehensive discussion about reproductive health behaviors in primary care settings (14). Responses included yes, no, maybe, do not know. For men, the OKQ was adapted to “do you want to father a child in the next year?” Participants were asked about their interest in co-located RSH services in general and in terms of specific programming using a six-point Likert-scale from “definitely would” to “definitely would not.”

#### Contraception

All participants were asked about their current contraceptive methods including both colloquial and brand name descriptions from a list of 12 common methods. Multiple responses were permitted. For analysis, contraceptive methods were separated into high, medium, and low efficacy tiers based on CDC criteria (15). Non-pregnant women reporting using high efficacy long-acting reversible contraceptives (LARCs) were asked about method use reasons and satisfaction. Satisfaction was rated with a five-point Likert-scale ranging from very satisfied to very dissatisfied. The same list of contraceptive methods was presented to determine contraceptive knowledge by method learned from a health professional. Participants reporting barriers to accessing contraceptives were asked to identify what barriers they faced from a list including (check all that apply): transportation, cost, time, availability, no local doctor, religious reasons, not a priority, and other (specify). Contraceptive decision-making agency captured who is responsible for contraceptive decisions, and contraceptive decision-making flexibility focused on whether contraceptive choices change based on the partner.

### Data Analysis Strategy

Variables were assessed for missingness and outliers (*z* scores >3.29) (16) and results presented with group means and stratified by gender and pregnancy status when relevant. A Chi-square analysis was used to test proportion of individuals who endorsed emergency contraceptive use, interest in ending a pregnancy early, pregnancy intention (OKQ, “Do you want to get pregnant in the next year?”), interest in RSH services, current contraceptive use (any), contraceptive method awareness, and if they have ever experienced barriers to accessing contraception by differences among gender and pregnancy status. A series of one-way analysis of variance (ANOVA) was run for contraceptive method awareness separated by efficacy by gender and pregnancy status. A Fisher’s Least Significant Difference *post hoc* test was used to determine significant differences by gender, pregnancy status and relevant variables. All analyses were conducted using SPSS v.25 (SPSS, Inc., Chicago, IL, United States) and criterion to reject the null hypothesis was set a *P* < 0.05.

## Results

### Participant Demographics

Adults (*N* = 225; 163 women, 62 men) provided informed consent. Complete data was available from 212 and was included in data analyses. The average participant was 33 years old (SD = 8.2), White (92.9%), with 12.5 years of education (SD = 1.8), and had Medicaid (92.0%). Twenty-nine women were currently pregnant, and one man’s partner was currently pregnant. Fourteen women had a hysterectomy, and 15 women were post-menopausal before the study began. See Table 1 for full demographics separated by gender and pregnancy status.

**TABLE 1:** Participant characteristics.

	Non-pregnant women (*n* = 126)	Pregnant women (*n* = 29)	Men (*n* = 57)	Total sample (*n* = 212)

Variable	*M* (SD)			
Age (years)	33.5 (8.2)	27.7 (6.0)	34.7 (8.2)	33.0 (8.2)
Education (years)	12.5 (1.8)	12.2 (1.3)	12.5 (2.1)	12.5 (1.8)

	***n* (%)**			

**Race**				
Non-White	12 (9.5)	1 (3.4)	2 (3.5)	15 (7.1)
White	114 (90.5)	28 (96.6)	55 (96.5)	197 (92.9)
**Ethnicity**				
Non-Hispanic/Latino	125 (99.2)	29 (100.0)	57 (100.0)	211 (99.5)
Hispanic/Latino	1 (0.8)	0 (0.0)	0 (0.0)	1 (0.5)
**Relationship**				
Never married	41 (32.5)	3 (10.3)	20 (35.1)	64 (30.2)
Married	23 (18.3)	6 (20.7)	15 (26.3)	44 (20.8)
Divorced	19 (15.1)	3 (10.3)	6 (10.5)	28 (13.2)
Separated	8 (6.3)	5 (17.2)	3 (5.3)	16 (7.5)
Living with a partner	27 (21.4)	11 (37.9)	13 (22.8)	51 (24.1)
Widowed	8 (6.3)	1 (3.4)	–	9 (4.2)
**Treatment group**				
Weekly	69 (54.8)	25 (86.2)	37 (64.9)	131 (61.8)
Bi-weekly	27 (21.4)	4 (13.8)	12 (21.1)	43 (20.3)
Monthly	30 (23.8)	–	8 (14.0)	38 (17.9)
Previous child (yes)	111 (88.1)	20 (69.0)	34 (59.6)	165 (77.8)
Tobacco use (yes)	110 (87.3)	25 (86.2)	49 (86.0)	184 (86.8)
Medicaid (yes)	120 (95.2)	27 (93.1)	48 (84.2)	195 (92.0)

### Reproductive Health

Most participants were sexually active in the past year (88.7%). Few participants (3.3%) reported same sex partners. Overall, 27.8% of participants (27.8% of non-pregnant women, 41.4% of pregnant women, and 21.1% of men’s partners) had ever used emergency contraception. Among the women, 65.7% of non-pregnant women (*n* = 35) and 33.3% of pregnant women (*n* = 12) reported using emergency contraception more than 2 times. There were no significant differences in ever using emergency contraception between non-pregnant women, pregnant women, and men [χ^2^(2) = 3.95, *P* = 0.138].

Endorsement for considering ending a pregnancy was low. Overall, 13.2% of participants (14.3% of non-pregnant women, 13.8% of pregnant women, and 10.5% of men) reported agreement with considering ending a pregnancy. There were no significant differences in agreeing with the statement, “I would consider ending/having my partner end a pregnancy early” between non-pregnant women, pregnant women, and men [χ^2^(4) = 5.08, *P* = 0.279].

#### Reproductive and Sexual Health Services in a Clinic

Reproductive and sexual health service interest is detailed in Table 2. Overall, 45.8% of participants (49.2% of non-pregnant women, 44.8% of pregnant women, and 38.6% of men) were interested in having general RSH services co-located at their clinic. There were no significant differences observed between gender and pregnancy status and interest in RSH services at their clinic [χ^2^(8) = 9.18, *P* = 0.327].

**TABLE 2:** Interest in RSH services at MOUD clinic.

	Non-pregnant women (*n* = 126)	Pregnant women (*n* = 29)	Men (*n* = 57)	Total sample (*n* = 212)

Variable	*n* (%)			
**Interest in RSH services**				
Definitely would	32 (25.4)	6 (20.7)	9 (15.8)	48 (22.6)
Probably would	30 (23.8)	7 (24.1)	13 (22.8)	50 (23.6)
Neutral	27 (21.4)	8 (27.6)	22 (38.6)	57 (26.9)
Probably would not	27 (21.4)	5 (17.2)	12 (21.1)	44 (20.8)
Definitely would not	10 (7.9)	3 (10.3)	1 (1.8)	14 (6.6)
**RSH services of interest**				
Contraceptive education and administration	48 (38.1)	17 (58.6)	16 (28.1)	81 (38.2)
STI/STD Testing	52 (41.3)	9 (31.0)	26 (45.6)	87 (41.0)
Regular physical exams	63 (50.0)	9 (31.0)	23 (40.4)	95 (44.8)
Pregnancy testing*	34 (27.0)	10 (34.5)	–	–
Ending a pregnancy*	9 (7.1)	2 (6.9)	–	–
Erectile function^+^	–	–	14 (24.6)	–
Premature ejaculation treatment^+^	–	–	10 (17.5)	–

*RSH, reproductive and sexual health.*

*Reproductive health addresses the reproductive processes, functions, and system at all stages of life (e.g., contraceptive counseling); sexual health is a state of physical, mental, and social well-being in relation to sexuality (e.g., sexual functioning).*

*Men were not asked questions about women specific services.

*^+^Women were not asked about male specific services.*

Many participants (44.8%) were interested in the hypothetical clinic based RSH services offering regular physical exams, 41.0% were interested in STD/STI testing, and 38.2% were interested in contraception education and administration. Gender-specific RSH service questions were asked. Women were interested in having pregnancy testing offered (27.0% of non-pregnant women and 34.5% of pregnant women). Men reported interest in services to help with erectile function (24.6%) and premature ejaculation (17.5%).

### Contraceptives

Contraceptive methods are detailed in Table 3.

**TABLE 3:** Contraceptive method awareness from a health professional.

	Non-pregnant women (*n* = 126)	Pregnant women (*n* = 29)	Men (*n* = 57)	Total sample (*n* = 212)		

Variable	*n* (%)					
**Awareness of high efficacy methods**						
Implant	54 (42.9)	13 (44.8)	16 (28.1)	83 (39.2)		
Intrauterine device	68 (54.0)	14 (48.3)	17 (29.8)	99 (46.7)		
Female/male sterilization	64 (50.8)	14 (48.3)	21 (36.8)	99 (46.7)		
**Awareness of medium efficacy methods**						
Oral contraceptive	101 (80.2)	22 (75.9)	27 (47.4)	150 (70.8)		
Ring	59 (46.8)	14 (48.3)	18 (31.6)	91 (42.9)		
Diaphragm	50 (39.7)	7 (24.1)	19 (33.3)	76 (35.8)		
Patch	66 (52.4)	15 (51.7)	13 (22.8)	94 (44.3)		
Injectable	78 (61.9)	20 (69.0)	18 (31.6)	116 (54.7)		
**Awareness of low efficacy methods**						
Condoms	90 (71.4)	19 (65.5)	40 (70.2)	149 (70.3)		
Withdrawal	35 (27.8)	9 (31.0)	17 (29.8)	61 (28.8)		
Fertility awareness	19 (15.1)	4 (13.8)	7 (12.3)	30 (14.2)		
Abstinence	70 (55.6)	21 (72.4)	37 (64.9)	128 (60.4)		
None	7 (5.6)	2 (6.9)	8 (14.0)	17 (8.0)		

	***M* (SD)**				***F***	***P***

Total contraceptive method awareness	4.4 (4.0)	6.0 (3.8)	5.9 (3.7)	5.5 (3.9)	3.59	0.029
High efficacy methods	0.9 (1.2)	1.5 (1.2)	1.4 (1.3)	1.3 (1.2)	3.90	0.022
Medium efficacy methods	1.7 (2.0)	2.8 (1.8)	2.7 (1.7)	2.5 (1.9)	7.88	0.001
Low efficacy methods	1.8 (1.2)	1.7 (1.3)	1.8 (1.2)	1.7 (1.3)	0.15	0.859

*Specifiers in the survey included: condoms (e.g., Trojans, rubbers, jimmies); injectable (depo injection; Provera); implant (Implanon, Nexplanon); intrauterine device (IUD); ring (NuvaRing); patch (Ortho Evra); oral contraceptive (the Pill); female/male sterilization (tubes tied; tubal ligation); fertility awareness (the rhythm method; menstrual cycle timing); Nexplanon was added as a descriptor for “implant” based on pilot testing.*

#### Current Contraceptive Method

Current use of contraception was low for non-pregnant women (30.9%) and men (17.9%). For non-pregnant women, the most common form of contraceptive method was female/male sterilization (18.6%) followed by the implant (7.2%), intrauterine device (7.2%), and oral contraceptive (7.2%). Four non-pregnant women reported using condoms as a contraceptive. The most common form of contraceptive methods reported by men was the condom (12.5%) followed by partner tubal ligation (8.9%).

Among participants who were not currently pregnant, had not had a hysterectomy, or were not post-menopausal, most non-pregnant women (69.1%) and men (82.0%) were not currently using contraception. Among this sub-sample of participants not currently using contraception, 76.1% of non-pregnant women and 91.3% of men were not interested in using contraception. That is, among reproductive-aged non-pregnant women and men in this sample, 76.1% of non-pregnant women (*n* = 51) and 91.3% of men (*n* = 42) were not interested in having a pregnancy over the next year, not currently using contraception, and not interested in using contraception.

#### Reason for and Satisfaction With Using High Efficacy Long-Acting Reversible Contraceptives

Among the nine non-pregnant women who reported using the implant, most non-pregnant women choose the implant for being reliable in preventing pregnancy (77.8%), ease of use (55.6%), based on a healthcare provider’s recommendation (55.6%), and for personal comfort (44.4%). Satisfaction for non-pregnant women who used the implant was high, with no non-pregnant women reporting any dissatisfaction, and the majority (66.7%) reported being very satisfied.

Among the eight non-pregnant women who reported using an IUD, most non-pregnant women chose an IUD for ease of use (87.5%), for being reliable in preventing pregnancy (87.5%), and for personal comfort (62.5%). Satisfaction for non-pregnant women who used an IUD was high, with no non-pregnant women reporting any dissatisfaction, and the majority (75.0%) reported being very satisfied.

#### Contraceptive Method Awareness

Contraceptive method awareness among 12 contraceptive methods is detailed in Table 3. Overall, the average participant was aware of 45.8% of contraceptive methods. Significant differences between non-pregnant women, pregnant women, and men by contraceptive method awareness were observed for total contraceptive method awareness (*F*_(2_,_209)_ = 3.59, *P* = 0.029). Non-pregnant women were aware of significantly more total contraceptive methods (*M* = 5.9; SD = 3.7) compared to men (*M* = 4.3; SD = 4.1). No significant differences were observed between the other groups and total contraceptive method awareness.

Significant differences were observed for high efficacy contraceptive method awareness (*F*_(2_,_209)_ = 3.90, *P* = 0.022). Non-pregnant women were aware of more high efficacy contraceptive methods (*M* = 1.5; SD = 1.2) compared to men (*M* = 0.9; SD = 1.2). No significant differences were observed between the other groups and high efficacy contraceptive method awareness.

Significant differences were observed for medium efficacy contraceptive method awareness (*F*_(2_,_209)_ = 7.88, *P* = 0.001). Non-pregnant women were aware of more medium efficacy contraceptive methods (*M* = 2.8; SD = 1.8) compared to men (*M* = 1.7; SD = 2.0). Pregnant women were aware of more medium efficacy contraceptive methods (*M* = 2.7; SD = 1.7) compared to men (*M* = 1.7; SD = 2.0). No significant differences were observed between the other groups and medium efficacy contraceptive method awareness.

No significant differences were observed between non-pregnant women, pregnant women, and men and low efficacy contraceptive method awareness (*F*_(2_,_209)_ = 0.15, *P* = 0.859).

#### Barriers to Accessing Contraception

Among the non-pregnant women who reported barriers to accessing contraception (13.5%), the most likely barriers selected were transportation (88.2%), cost (52.9%), availability (52.9%), and time (47.1%). Among the pregnant women (31.0%) and men (10.5%) who reported barriers to accessing contraception, no theme emerged regarding specific barriers to accessing contraception from the list provided. However, pregnant women (31.0%) were roughly three times more likely than non-pregnant women (13.5%) and men (10.5%) to have experienced a barrier to accessing contraception [χ^2^(2) = 6.93, *P* = 0.031].

#### Contraceptive Decision-Making Agency and Flexibility

**FIGURE 1:**
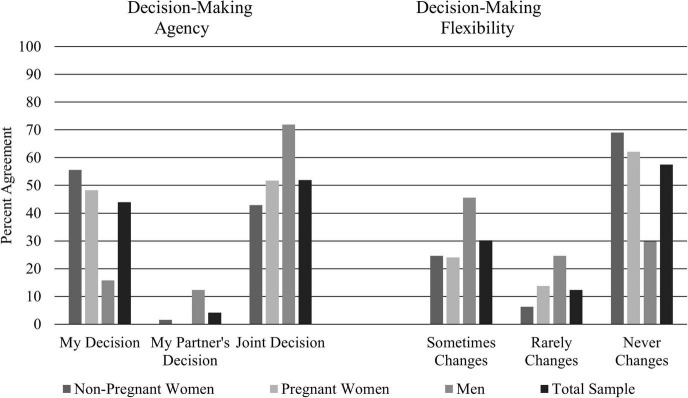
Contraceptive decision-making agency and flexibility. Significant differences were observed between women and pregnant women and men for both decision-making agency and decision-making flexibility. Contraceptive decision-making agency was assessed with the question: “Whose decision is it to use birth control?” Contraceptive decision-making flexibility was assessed immediately after the contraceptive decision-making agency with the question: “Does the decision change depending on who the partner is?”

Contraceptive decision-making agency and flexibility is detailed in Figure 1. Contraceptive decision-making agency was significantly different between non-pregnant women, pregnant women, and men [χ^2^(4) = 32.7, *P* < 0.001]. Non-pregnant women (55.6%) and pregnant women (48.3%) were more likely to respond that contraceptive use was “my decision” compared to men (15.8%). Men were significantly more likely to respond that contraceptive use was “my partner’s decision” (12.3%) and “both our decision” (71.9%) than non-pregnant women and pregnant women.

Contraceptive use flexibility was significantly different between non-pregnant women, pregnant women, and men [χ^2^(4) = 27.4, *P* < 0.001]. Non-pregnant women (69.0%) and pregnant women (62.1%) were more likely to respond that contraceptive use never changes depending on whom the partner is compared to men (29.8%). Men were significantly more likely to respond that contraceptive use was rarely (24.6%) and sometimes (45.6%) flexible depending on who the partner is.

## Discussion

To our knowledge, this is among the first empirical reports to document a range of RSH behaviors for both women *and* men with OUD receiving MOUD. By including women and men, we were able to understand some unique interests for each population. Overall interest in co-located RSH was high among both women and men. This contrasts with previous research that found limited co-located RSH services in OUD treatment facilities (2), suggesting a desired and unmet patient need.

### Interest in Co-located Reproductive and Sexual Health Service

This work extends Black and associates (5) previous findings that 24.5% of women indicated a preference for women’s health services to be integrated with MOUD. In the present study, approximately 40% of women and men were interested in same-day, co-located RSH services including contraception. Notably, the Black and associates study provided various settings that could accommodate RSH services (e.g., general practitioners and sexual health clinics) and was not assessing specific level of interest in co-located services at a MOUD treatment facility. In contrast, the present study was specific to co-located RSH services at a MOUD treatment facility.

Importantly, there may be a greater need for and interest in integrated services in rural populations. For example, the present study had a mean travel time of over 1 h, and most patients attend treatment for over 3 h per week. Interest in co-located services may be a necessity more than a matter of convenience for this sample. Additionally, the most common barriers faced when trying to access contraception were transportation, cost, and time. Co-located RSH services are one way to address these barriers. Nationally, the degree to which RSH services have been integrated into OUD treatment facilities is low; however, robust national data are lacking (2).

While the RSH service umbrella is large, our findings highlight often overlooked elements of comprehensive care. For example, both women and men were interested in STI/STD testing at a co-located RSH clinic. There was also interest in male specific services such those that focus on premature ejaculation treatments and erectile function. This aligns with potential sexual dysfunction associated with opioid use (17–20); however, treatment for these related conditions are often overlooked as part of comprehensive care or focused RSH services. While emergency contraception was not included as a potential “service” to be offered at clinics in our survey, between 27 and 41% of women reported its use suggesting that questions about emergency contraception should be included in future research. Additionally, consideration of ending a pregnancy early was 13.2% across the complete sample suggesting that ethical counseling related to ending a pregnancy grounded in respect for patient autonomy should also be the standard of care at OUD clinics that integrate RSH services.

### Contraceptive Knowledge and Use

Participants in this sample were also interested in contraceptives being included in co-located RSH services. Although interest in co-located contraceptive counseling was high, current contraceptive use and knowledge about contraception was low, in both men and women. Knowledge of high and medium efficacy approaches was low overall but lower among men than women. Both groups knew similar amounts of low efficacy approaches. The lack of knowledge regarding high efficacy LARCs may contribute to both the high rates of unintended pregnancy and ambivalence around contraceptive use. Women who used high-efficacy LARCs (e.g., IUD) reported more satisfaction with their use, few women had previous knowledge about high-efficacy LARCs which may explain why only 46% of participants were interested in contraceptive services at the clinic and be a target of future interventions.

Among the non-pregnant sub-sample, most women and men were not interested in having a pregnancy over the next year; however, they were not currently using contraception, and were not interested in using contraceptives. This counterintuitive finding can be understood in several ways. First, while counterintuitive that individuals who do not want a pregnancy in the next year are also not engaging in contraceptive use, low knowledge of high efficacy contraceptives may be one explanation borne out in our findings. Second, it is worth noting that our sample is on the low end of condom use (4.3% of women and 12.5% of men) compared to the Terplan and associates (8) review of women with substance use disorders (range of 3–87%) and the approximately 9% of reproductive-age women in the United States who reported male condom use between 2015 and 2017 (21). While low, condom use in our sample may be accurate, it is also possible that because the question assessed “birth control” use, there may have been some underreporting due to confusion. It is possible that condoms are not always thought of as a form of “birth control” and are more associated with HIV and STI/STD prevention, especially in OUD patient populations where contraceptive materials are often tailored toward the dual role of HIV prevention and contraception. Third, low interest in contraceptive approaches may be due to low knowledge of high efficacy approaches from health professionals that require less daily maintenance and are relatively new (e.g., implants and IUDs) compared to approaches that need constant attention and are more commonly used in the United States (e.g., the Pill and condoms). Indeed, among participants in our sample who reported use of high efficacy LARCs, satisfaction was high. They were preferred for their ease of use (55.6–87.5%), reliably preventing pregnancies (77.8–87.5%), and personal comfort (44.4–62.5%) for implants and IUDs, respectively. While LARC use was low in this sample, this indicates that these methods can be acceptable to women with OUD, and knowledge may be a barrier to more widespread use.

Previous research has shown that high-efficacy contraceptive use is low among patients with an OUD (8). While evidence-based contraception counseling methods can help increase knowledge about newer contraceptives (22), education alone may not ultimately lead to new behaviors unless other barriers are addressed, such as access that can occur through co-located services. Contraceptive decision-making does not happen in a vacuum. Women were more likely to report contraceptive use as their decision and that this decision is not flexible. In contrast, men were more likely to report that it is a joint decision and that there is some flexibility in contraceptive use depending on the partner. These discordances between knowledge and decision-making by gender could make for challenging discussions between partners regarding contraceptive method choice. As such, educational initiatives aimed at contraception should be inclusive of men as they may play a role in contraceptive decision making and are less likely to be familiar with high efficacy approaches. Recent research shows that providing either face-to-face or computerized RSH services using a shared decision making approach, between provider and patient, to non-pregnant women receiving MOUD hold promise for increasing both decision making and follow through on a contraceptive practice decision compared to usual care (6). Increasing access to person-centered contraceptive counseling through co-located RSH services can help better fulfill the health needs of this patient population and have been shown to be cost effective (23).

### Limitations of the Current Study

This study is not without limitations. Our study was at a single site in which may limit generalizability of our findings to OUD patients receiving MOUD in different geographic locations with different access to RSH services. While sexual health applies to all participants in the study, reproductive health may not. Future research may consider separating out these domains. In this study, gender was only presented as a binary choice (male and female) and did not include the full spectrum of potential gender identities. This was a cross-section survey without follow-up; thus, causality could not be determined. Additionally, while the study included men and women, it was only piloted in women because there were relatively few men attending this clinic. Lastly, our sample was 92.9% white and primarily women. While this is representative of the clinic, this is not representative of OUD. Future work should address these limitations in more diverse samples. Despite this weakness, this study has unique strengths. It is the first to document interest in the co-located RSH services into addiction treatment in women and men and broadly characterize contraceptive knowledge and decision making.

## Conclusion

Based on these findings, we recommend that both contraceptive counseling and provision of contraceptives be provided at MOUD programs due to the co-occurring low knowledge of contraceptive options and low utilization of high-efficacy, reversible methods. Most MOUD clinics have staff that could be trained to provide most LARC methods. If a patient receives their MOUD at their primary care providers office or a federally qualified health center, then these services are already available; however, most patients receive their care at a MOUD clinic, highlighting the need to co-locate RSH services in traditional treatment settings.

Co-located RSH and MOUD services are beneficial and a substantial minority of both women *and* men in our study are interested in various co-located RSH services. Co-located RSH and MOUD services are especially important in rural communities with limited access to these services. Knowledge of contraceptive methods and use of contraception was low. Contraceptive decisions varied based on interpersonal dynamics in our participants’ relationships. These factors underscores the importance of assessing the RSH needs of both men and women in OUD treatment. Taken together, providing RSH services may allow for increased RSH education, increased uptake of contraceptive methods, and healthier life outcomes. This research suggests that including RSH services would not only address an un-met need but would move addiction treatment to be more holistic.

## Data Availability Statement

The raw data supporting the conclusions of this article will be made available by the authors, without undue reservation.

## Ethics Statement

The studies involving human participants were reviewed and approved by the West Virginia University Institution Review Board. The patients/participants provided their written informed consent to participate in this study.

## Author Contributions

All authors were involved in the conceptualization and methodology of the work described in this manuscript, contributed substantively to the content of the manuscript, and review and editing of the manuscript. JS, LL, and JP handled the project management. JS led analyses and manuscript writing. All authors have approved the final version of the manuscript.

## Author Disclaimer

The content is solely the responsibility of the authors and does not necessarily represent the official views of the NIH.

## Conflict of Interest

The authors declare that the research was conducted in the absence of any commercial or financial relationships that could be construed as a potential conflict of interest.

## Publisher’s Note

All claims expressed in this article are solely those of the authors and do not necessarily represent those of their affiliated organizations, or those of the publisher, the editors and the reviewers. Any product that may be evaluated in this article, or claim that may be made by its manufacturer, is not guaranteed or endorsed by the publisher.
